# Development and Characterization of Polypropylene Waste from Personal Protective Equipment (PPE)-Derived Char-Filled Sugar Palm Starch Biocomposite Briquettes

**DOI:** 10.3390/polym13111707

**Published:** 2021-05-24

**Authors:** M. M. Harussani, S. M. Sapuan, Umer Rashid, A. Khalina

**Affiliations:** 1 Advanced Engineering Materials and Composites Research Centre (AEMC), Department of Mechanical and Manufacturing Engineering, Universiti Putra Malaysia, UPM Serdang 43400, Selangor, Malaysia; mmharussani17@gmail.com; 2 Laboratory of Biocomposite Technology, Institute of Tropical Forestry and Forest Products (INTROP), Universiti Putra Malaysia, UPM Serdang 43400, Selangor, Malaysia; khalina@upm.edu.my; 3 Institute of Advanced Technology (ITMA), Universiti Putra Malaysia (UPM), Serdang 43400, Selangor, Malaysia

**Keywords:** biopolymer composites, PPE, polypropylene, COVID-19 related waste, char briquette, sugar palm starch, pyrolysis

## Abstract

Slow pyrolysis using a batch reactor at 450 °C was applied to the polypropylene (PP) powder derived from Coronavirus Disease 2019 (COVID-19) isolation gown waste to yield char briquettes, using sugar palm starch (SPS) and a manual hydraulic press. These studies are significant because of reductions in plastic waste from the preparation of barbecue coal due to environmental sustainability. The results presented here include the physical, morphological, thermal, combustion, and mechanical properties of char when reinforced with various percentages of SPS loadings (0, 10, 20, 30, and 40%), which act as a matrix/binder to produce char/sugar palm starch (C/SPS) composites. The physical and morphological characteristics of C/SPS composites were determined using Fourier transform infrared (FTIR) and field emission scanning electron microscopy (FESEM). On the other hand, the thermal and combustion properties of the C/SPS briquettes were studied via thermogravimetric and bomb calorimeter analysis. The results show that the compressive strength of the briquettes increased as the SPS loading increased, whereas the higher heating values (HHV) reduced. The findings indicate that C-80/SPS-20 briquettes presented excellent combustion characteristics (1,761,430 J/g) with satisfactory mechanical strength (1.463 MPa) in the compression test. Thus, C-80/SPS-20 briquettes are the most suitable composites for domestic and commercial uses.

## 1. Introduction

The Ministry of Health (MOH) Malaysia used personal protective equipment (PPE) made from non-woven fabric polypropylene (PP) to fabricate masks, isolation gowns, hair nets, and shoe covers [1]. Currently, 59 million units of PPE are being used by health staff under the MOH per month. As a result, approximately 2.124 billion units had been utilized over the course of two months [2]. Thus, high utilization of plastic products will lead to high production of environmental plastic waste. Generally, polypropylene waste (PP) takes 20–30 years to decay completely. These plastics contain additive materials—including colorants, plasticizers, and stabilizers—composed mainly of cadmium and lead [3], which are harmful to the environment. As mentioned by Verma et al. [4], waste plastics can make up as much as 28% of overall cadmium in urban solid waste. Thus, plastic waste can emit significant contaminants into the atmosphere when burned openly. Due to these harmful impacts of plastic waste and its management practices on the environment, more environmentally sustainable methods for plastic waste disposal should be created.

**Figure 1:**
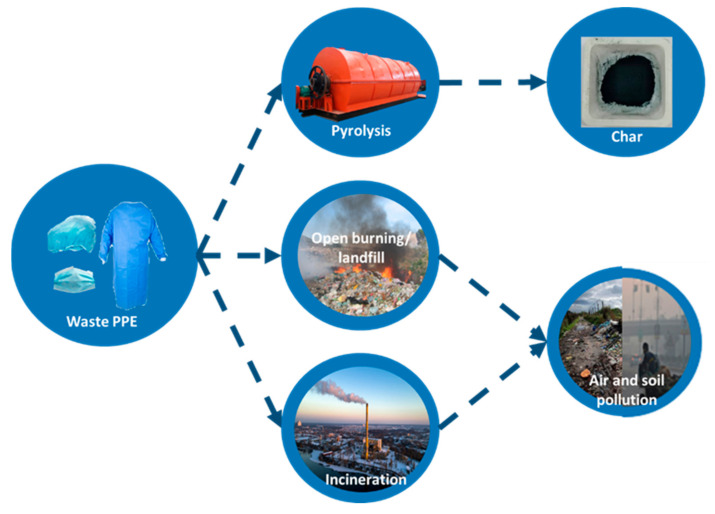
Pyrolysis of waste plastic to char product.

There are significant variations between existing incinerator practice and the proposed pyrolysis method. Incineration of plastic waste primarily produces carbon dioxide, water, and unburned material that exists in the bottom ash—called micro plastics [5]—while waste pyrolysis plants primarily produce combustible, low molecular weight compounds largely composed of gaseous substances such as hydrogen, nitrogen, and carbon monoxide; liquid substances such as methanol, acetone, acetic acid, acetaldehyde, and other organic matter, as well as tar, solvent oil, and other solid substances; and solid products including coke, char, and carbon black [6,7,8]. Figure 1 shows the schematic illustrations of pyrolysis and current practices.

Pyrolysis is the thermochemical decomposition of materials, such as waste plastics, at high temperatures in a deoxygenated environment [9]. Plastic waste decomposes into organic compounds with a lower molecular weight during this phase. In general, the products will be composed of solid substances as well as a variety of condensable and non-condensable volatile oil and gas products. Product yields are largely determined by the waste’s physical and chemical characteristics, as well as other pyrolysis conditions—including pyrolysis temperature, friction, heating rate, and residence time [10]. Pyrolysis is a low-pollution process. Moreover, it has been mentioned in previous works that pyrolysis leads to high waste conversion into various valuable products [11,12]. As such, it will have excellent applications in converting plastic waste into usable goods [8,13,14]. For instance, there are only a few articles in the literature on the pyrolysis of plastic waste and the conversion of the solid pyrolysis materials—such as char—into briquettes with aid from binder materials.

Plastic and bio-waste materials, including polythene bags and maize husks, were pyrolysed into char and mixed with various types of binders. In the work of Nwabue et al. [15], coal was mixed with limestone dust, cassava flour, and laterite for the solid fuel briquette production. Bio-char briquettes with 4.37 MPa of mechanical strength and a calorific value of 20 MJ/kg were fabricated. The composition of various binders with carbonized plastic waste led to smokeless and efficient combustibility. Citrasari et al. [16] studied charcoal briquettes made from carbonized sludge and leather cassava mixed with tapioca flour. Briquettes with 40% carbonated leather composition showed low mechanical strength, and calorific values of 7.68 MJ/kg. On the other hand, Zannikos et al. [17] investigated the combustion properties of various compositions of solid fuel briquettes derived from waste plastic of PET and sawdust. Similar work was conducted by Garrido et al. [18], who produced fuel briquettes from sawdust and pelletized date palm trunk with plastic waste. From the literature, we can conclude that plastic waste-derived fuel briquettes exhibited low calorific values when compared to char briquettes made from biomass waste [19,20,21,22] (see Table 1). As reported by Zanella et al. [19], slow pyrolysis of orange bagasse at 450 °C produced orange charcoal, which mixed directly with corn starch. In briquette biocomposites preparation, natural binder such as corn, and cassava starch were commonly used [23,24]. Corn starch loading of 15% inside the briquette led to a high calorific value of 26 MJ/kg, with 2.1 MPa mechanical strength. The converting of various waste products to hydrocarbon mixtures via pyrolysis has attracted much attention, because this process might allow for the reduction of the amount of waste, the recovery of chemicals, and the replacement of other fuels [25].

Some studies on the calorific value of char briquettes are summarized in Table 1. From the existing literature, only a few works on plastic pyrolysis char were evaluated for solid fuel briquettes, due to the limited char yields from laboratory-scale experiments. Thus, there was no pre-existing scientific study in which solid product char obtained from pyrolysis of PP waste was used as briquette char. Pyrolysis studies related to PP waste are generally catalytic pyrolysis studies, and only pyrolysis product properties were examined in these studies [26]. Conversely, our work presents the pyrolysis of real PP waste and the preparation of briquettes from pyrolysis char. In this study, PP waste derived from COVID-19 isolation gowns was pyrolysed in the batch pyrolysis reactor, and the influence of binder varieties on the quality of the resulting briquettes was investigated.

## 2. Materials and Methods

### 2.1. Materials

Polypropylene (PP) powder was obtained from pulverised, disinfected COVID-19 isolation gowns collected from the university healthcare centre. The collected PPE was shredded using a FRITSCH Universal Cutting Mill (PULVERISETTE 19, Idar-Oberstein, Germany) into approximately 0.25 mm of PP powder. The smaller size of the feed samples led to the complete carbonization of the plastic waste at the end of the process. The surface moisture was removed from the plastic powder via the oven-drying method and, thus, the yield of the solid product was improved, and the residence time was reduced. This drying approach was done in a forced-air oven (Lichen, Zhejiang, China) at 105 °C. The powder samples were stored in an alumina boat prior to pyrolysis.

Sugar palm starch (SPS) was extracted from sugar palm trees planted at the village of Kuala Jempol, located in Negeri Sembilan, Malaysia. SPS used in this study was composed of 37.8% amylose and 62.2% amylopectin.

### 2.2. Slow Pyrolysis Process and Char Preparation

Slow pyrolysis was performed in order to thermally decompose the PP powder samples using laboratory-scale batch reactor (Fisher Scientific, Loughborough, Leicestershire, UK) with a specific pyrolysis temperature of 450 °C, heating rate of 3 °C/min, and 1.5 atm pressure, in a deoxygenated environment. Twenty grams of PP powder was filed into alumina boats and loaded into a 12-mm internal diameter horizontal ceramic tube furnace of the batch reactor. Purified nitrogen gas (99.9992%) was purged into the reactor, at flow rate of 1.5 cc/min. The next step, once the plastic waste powder had been inserted into the reactor chamber, was the heating of the chamber to the specified pyrolytic temperature, which was then maintained for 5 h.

The solid char yield was obtained by following Equation (1):

(1) $$Y_{char}=\frac{m_{product}}{m_{waste}}\times 100$$

where $Y_{char}$ is the char yield percentage collected via the slow pyrolysis process, $m_{waste}$ is the initial mass of the plastic waste powder, and $m_{product}$ is the mass of yielded char products.

### 2.3. Char Briquette Preparation

**Figure 2:**
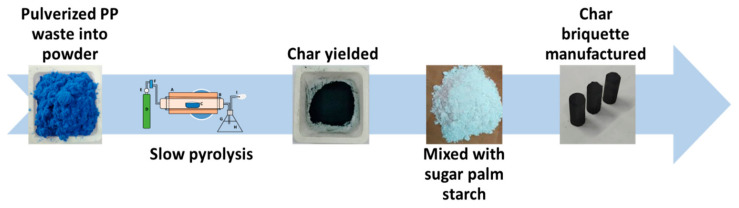
Flow of the process of manufacturing char briquettes (C/SPS).

The sifted charcoal particles were mixed with sugar palm starch at ratios of 10%, 20%, 30%, and 40%. The sugar palm starch was cooked in water to make glue with a ratio of 1:3 at a temperature of 80 °C. This starch binder was combined with the fine raw materials to form a homogeneous mixture, which was then poured into a mould and subjected to a predetermined pressure. The hydraulic press (TOYO, Kuala Lumpur, Malaysia) that poured the sample mixture into the mould had a pressure of 1.5 tonnes under ambient temperature. The mould was made up of three parts: the frame, a 12.7 mm-diameter cylindrical tube, and a solid cylinder that served as a piston to apply pressure to the raw materials in the cylindrical tube. After the briquettes had taken shape, they were dried in an oven at 60 °C for 24 h. Since high water content in briquettes promotes fungal growth, this drying was intended to reduce the water content, which increases during the moulding process [19]. Figure 2 shows the schematic illustration of the process flow for manufacturing char briquettes. The next steps are sample characterizations and mechanical testing.

### 2.4. Biocomposite Briquette Characterizations

The thermal properties of the char briquettes were evaluated out using Mettler Toledo’s TGA-DSC HT 3 equipment (Mettler Toledo, Shah Alam, Selangor, Malaysia) to measure the changes in the properties of the char under elevated temperatures via thermal decomposition. TGA was carried out using a temperature range of 25–600 °C, with a 10 °C/min heating rate. Whereas proximate analysis was evaluated by keeping the samples in an oven with a forced circulation of air at 105 °C until their constant weight was achieved, the humidity level was calculated by weight loss via the gravimetric process. On the other hand, the elementary and morphological analyses were carried out using a Max 20 Energy-Dispersive X-Ray (EDX) (Oxford Instruments, Oxford, UK) and field emission scanning electron microscopy (FESEM) using Nova NanoSEM 230 FESEM (FEI, Sydney, Australia). Various high magnifications were used in order to get clearer micro-images of the samples. Fourier transform infrared (FTIR) spectroscopy was based on a 400–4000 cm^−1^ attenuated total reflective (ATR) method, which provides the identification of functional groups in the briquette samples. A Nicolet 6700 FTIR Spectrometer (Thermo Fisher Scientific, Waltham, MA, USA) was used. The calorific values of the C/SPS briquettes were evaluated via bomb calorimeter, Parr 1341 Oxygen Bomb Calorimeter (Parr Instrument Company, Moline, IL, USA) in accordance with ASTM International Standard E711-87 [32]. A bomb calorimeter was used to calculate the higher heating values (HHV). This method measures the heat released by determining the temperature difference between the sample placed in the container before and after the full combustion phase.

### 2.5. Density and Compressive Strength Analysis

**Figure 3:**
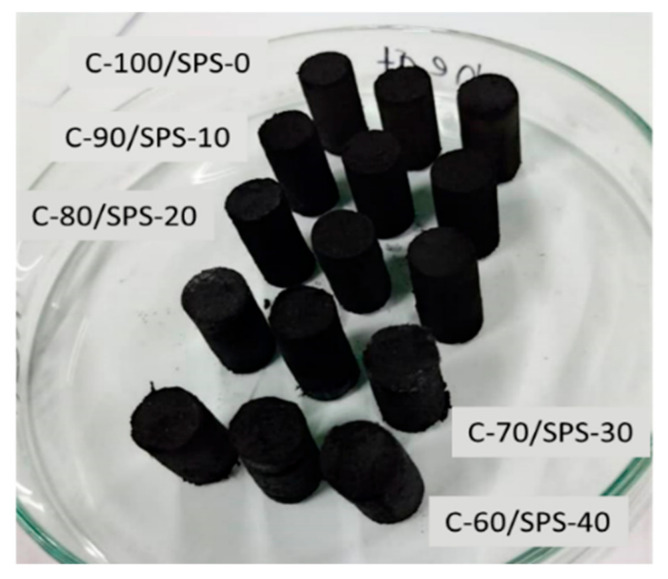
Biocomposite char briquettes via compression moulding.

The density of the charcoal briquettes was determined 72 h after compaction using an analytical balance and a digital calliper. The density results were calculated using the mass-to-volume ratio of each briquette. Figure 3 represents the fabricated briquettes in cylindrical shapes. The mechanical strength of charcoal was calculated using a compression test. An Instron 3382 universal testing machine (High Wycombe, UK) was used to determine compressive strength in accordance with ASTM D695 [33]. The modification and starting speeds were both 0.3 cm min^−1^. The yield and the compressive strength of the samples were measured using this test.

### 2.6. Statistical Analysis

The analysis of variance (ANOVA) on the obtained experimental results of compressive strength and density was performed in SPSS software. Duncan’s test was employed to conduct a mean comparison at a 0.05 level of significance (*p* ≤ 0.05).

## 3. Results and Discussion

### 3.1. Morphological and Elemental Analysis

**Figure 4:**
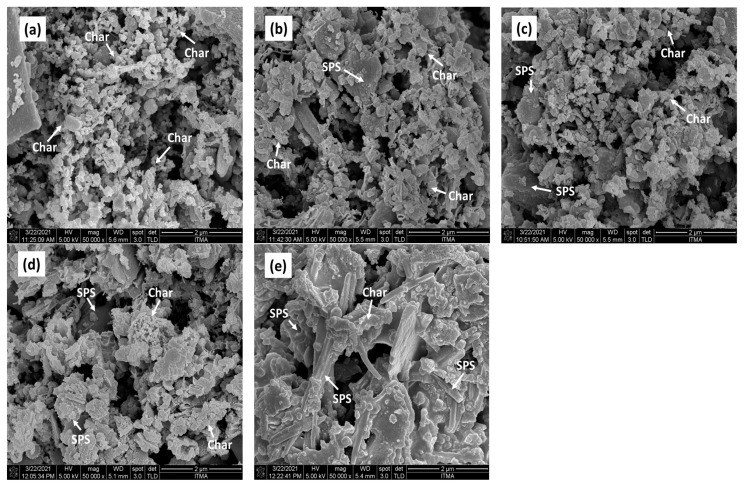
FESEM images of (**a**) C-100/SPS-0, (**b**) C-90/SPS-10, (**c**) C-80/SPS-20, (**d**) C-70/SPS-30, and (**e**) C-60/SPS-40.

The microstructural and morphological analysis of the char/sugar palm starch (C/SPS) composites was conducted using the FESEM method. The mechanical structure’s relation to the mechanical strength of the briquettes was determined via morphological analysis. Figure 4 depicts the morphological analysis of the C/SPS briquettes at the high magnification of 50k×. Based on the observations, it was found that the microstructure of the briquettes is apparently finely textured for C-100/SPS-0, but has a coarse and rigid structure for C-90/SPS-10, C-80/SPS-20, C-70/SPS-30, and C-60/SPS-40 briquettes. This is due to the presence of SPS as the binder inside the composites, which can be observed from the increased amount of SPS, as shown in Figure 4 [19,34]. This microstructure proves that good bonding of the fine particles and decreased porosity were obtained for the briquettes with SPS loading compared to the neat ones.

**Figure 5:**
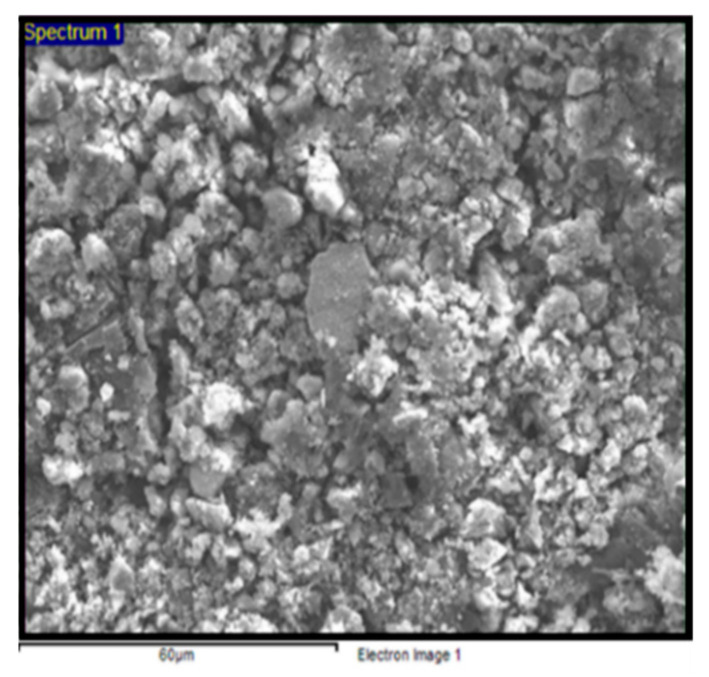
Electron image for EDX analysis.

**Figure 6:**
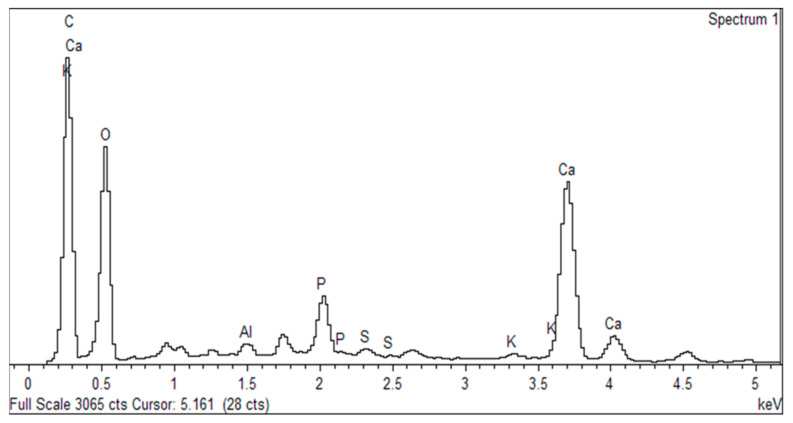
EDX analysis spectrum of C/SPS briquettes.

**Figure 7:**
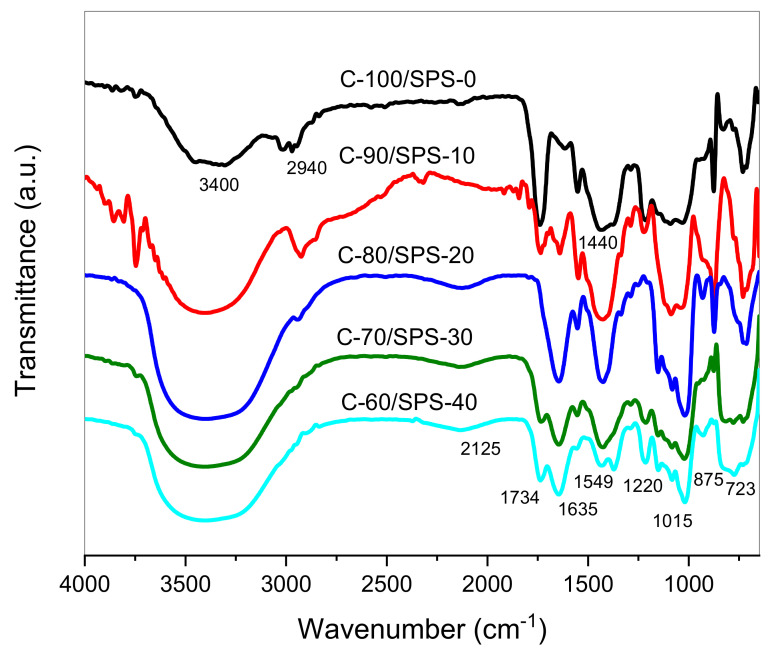
FTIR spectra for C/SPS briquettes.

**Figure 8:**
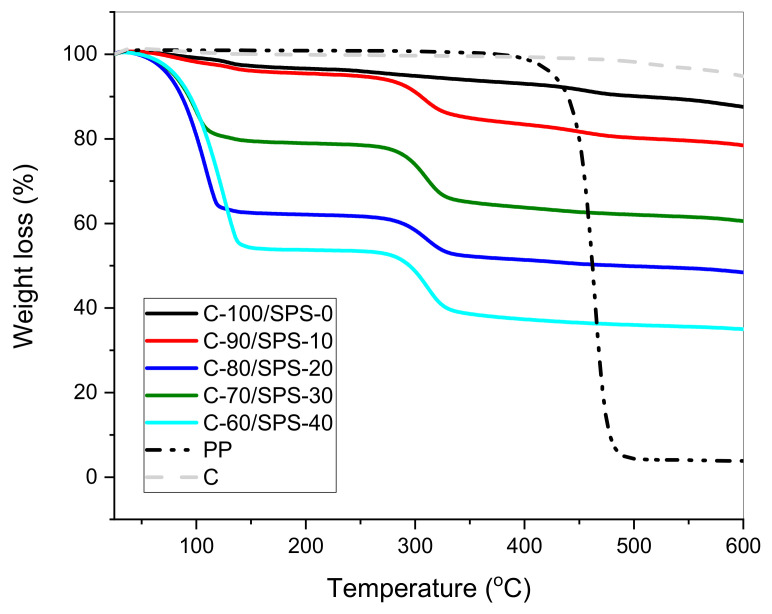
TGA curves for the raw PP, yielded char, and char briquettes within the temperature range of 25–600 °C.

EDX analysis was carried out in order to study the elemental properties of the char briquettes as shown in Figure 5. The contents of the char briquettes, generated from the EDX results, are displayed in Table 2. From Figure 6, the dominant element in the C/SPS composites is carbon (43.28%), although the atomic percentage of oxygen is higher, at 46.14%. This is due to the high moisture content within the briquettes; thus, this finding is in line with the FTIR spectra (see Figure 7) and TG analysis (see Figure 8). Carbon and oxygen are accompanied by other low weight percentage elements, including calcium (7.91%), phosphorus (1.91%), sulphur (0.25%), and aluminium (0.34%). Table 2 shows the lists of other elements. Based on the work of Basu [35], P, Al, Ca, and K are the primary components of the ash that formed during pyrolysis as parts of the char yielded.

### 3.2. Proximate Analysis

The microstructural Table 3 shows the proximate analysis of the C/SPSs fabricated with various amounts of binder loading, which follows Equation (2). The char briquette samples were characterized by high moisture and fixed carbon contents and a low ash content.

(2) $$Fixed\;carbon\;\left(\%\right)=100-Volatile\;Matter-Ash\;Content$$

The study showed that the moisture content within the briquettes increased with the increasing SPS loading, especially for C-70/SPS-30 and C-60/SPS-40. This happened due to the existence of high amounts of starch, which leads to a high rate of moisture absorption, attributed to its hydrophilic nature [36,37]. It also can be seen in Table 3 that the composites with SPS loading have higher volatile content, and so reduced the fixed carbon content compared to briquettes with 0% SPS content, with weight percentages of 4.88 wt.% and 88.75 wt.%, respectively. This observation is in agreement with the work of Zanella et al. [19]. On the other hand, it can be seen that the briquettes showed approximately the same ash content irrespective of the increasing binder loading. This occurred as a result of the binder’s lack of ash in its formulation, instead relying on volatiles [19,21]. Regarding the fixed carbon content, it is noted that the briquettes exhibited high amounts of fixed carbon, which matches the literature related to briquettes from biomass and plastic waste. Tienne et al. [20] obtained only 68.75 wt.% of fixed carbon content in their orange peel charcoal, whereas only 42.92 wt.% of fixed carbon content in orange charcoal with corn starch was observed by Zanella et al. [19]. Conversely, Onukak et al. [21] yielded a high fixed carbon content of 92.38 wt.% for their biomass briquettes from pre-treated tannery solid waste. In addition, the results of high fixed carbon content equate to high calorific values of the briquettes, as shown in Table 4.

### 3.3. Functional Group Analysis

ATR–FTIR spectra of PP char reinforced with SPS briquettes C-100/SPS-0, C-90/SPS-10, C-80/SPS-20, C-70/SPS-30, and C-60/SPS-40 show consistency in their functionality and characteristics, as shown in Figure 7 IR spectra for the briquettes represented changes in the bands occurring in the proximity of 4000 and 600 cm^−1^, which then explained the qualitative and quantitative analyses for the identified chemical bonds and chemical compounds in a wide range of capacities. The changes that happened were mainly due to increasing starch content within the samples. Broad absorption bands with strong intensity at a range of 3400–3300 cm^−1^ corresponded to the hydroxyl –OH groups’ stretching and bending, which caused the physisorption of moisture adsorbed onto the surface of the briquettes [38]. The C–H stretching of the aliphatic groups methyl, methylene, and methane existed in the char results in a narrow band at 2940 cm^−1^. The stretching of the C=O conjugated and unconjugated (carbonyl/carboxyl) bonds of carboxylic acids within the starch was consistent with the peak at 1734 cm^-1^. The area of the spectrum between 1400 and 1500 cm^−1^, or 1550 and 1600 cm^−1^, demonstrated the presence of C=C stretching vibrations due to aromatic rings, and an intense absorption peak of stretching vibration from the C=C bonds of alkenes arises in the spectral range of 1600–1680 cm^−1^ [39,40]. In the briquettes spectrum, the high intensity absorption peaks were discovered at 1429 cm^−1^ and 1560 cm^−1^ (C=C), and 1640 cm^−1^, respectively. Vibrations in C–O, C=C, and C–C–O within the char and SPS provide one of the most pronounced bands in the area, between 1031 and 1034 cm^−1^. Sogancioglu et al. [41] mentioned that the distinct peaks in the spectral regions between 800 and 900 cm^−1^, and 700 and 800 cm^−1^ represent p-disubstituted benzene aromatic C–H and alkene groups. The distinct peaks at 876 cm^−1^ and 723 cm^−1^ were observed from the IR spectra, respectively. Conclusively, aliphatic bands and alkene bands were reduced greatly with the increase in SPS content. This was due to successful binding of the char materials with the starch, which then produced rigid structures and high mechanical strength, as represented in Table 5.

### 3.4. Thermogravimetric Analysis

**Figure 9:**
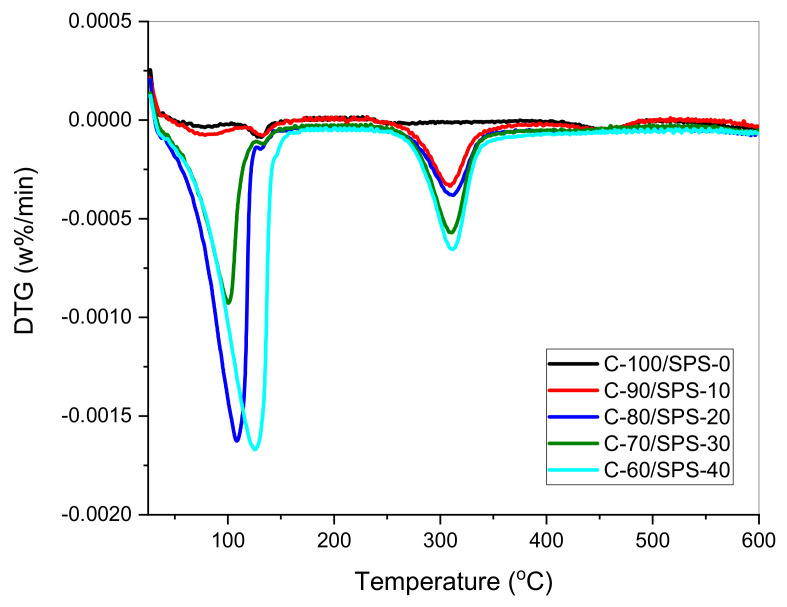
DTG curves for C/SPS briquettes.

TGA is a standard method for the measurement of weight loss with respect to time or temperature. The thermal stability of C/SPS char briquettes with various loadings of SPS was ascertained by using TG and DTG curves, as shown in Figure 8 and Figure 9, respectively, under non-isothermal conditions, with a heating rate of 10 °C/min, in the temperature range 25–600 °C. From Figure 8, raw PP shows a single degradation step. PP degradation began at a lower temperature of 400 °C, due to the fact that each PP polymer chain carbon atom composed of the polymer branching is tertiary carbon. The decomposition of PP chain branches and double-bonded backbones with a weight loss of 97 % occurred at temperature range of 400–500 °C. Whereas the residue after 550 °C was annotated as solid product of carbon-rich char [42,43].

On the other hand, the first stage of thermal decomposition of char briquettes C-100/SPS-0, C-90/SPS-10, C-80/SPS-20, C-70/SPS-30, and C-60/SPS-40, in the temperature range of 25–140 °C, with a weight loss of approximately 1.33, 2.36, 18.53, 36.52, and 44.67%, respectively, was attributed to the removal of adsorbed water molecules on the surface of the char briquettes. Similar reactions happened towards un-briquetted char sample, C, as shown in Figure 8. The weight loss was due to the presence of SPS, which enhanced the hydrophilic nature of the composites [44]. The second phase, within the temperature range of 250–600 °C, was associated with the condensation reaction of hydrocarbons and the formation of coke. This phase is an active phase of pyrolysis, characterized by a large weight loss of approximately 15 %, where a great quantity of volatile matter and gases is generated. The degradation occurred as a result of (1) the formation of free radicals during the initiation of degradation of the polymer chains, and (2) the diffusion of volatile degradation products [45]. In addition, the DTG curves also define the characteristics of two phases of char degradation. Figure 9 shows the DTG curves for char briquettes C-100/SPS-0, C-90/SPS-10, C-80/SPS-20, C-70/SPS-30, and C-60/SPS-40 under non-isothermal conditions, with a heating rate of 10 °C/min in the temperature range 25–600 °C. The weight loss rate of the char briquettes reduces in intensity with the increase of SPS loading.

**Figure 10:**
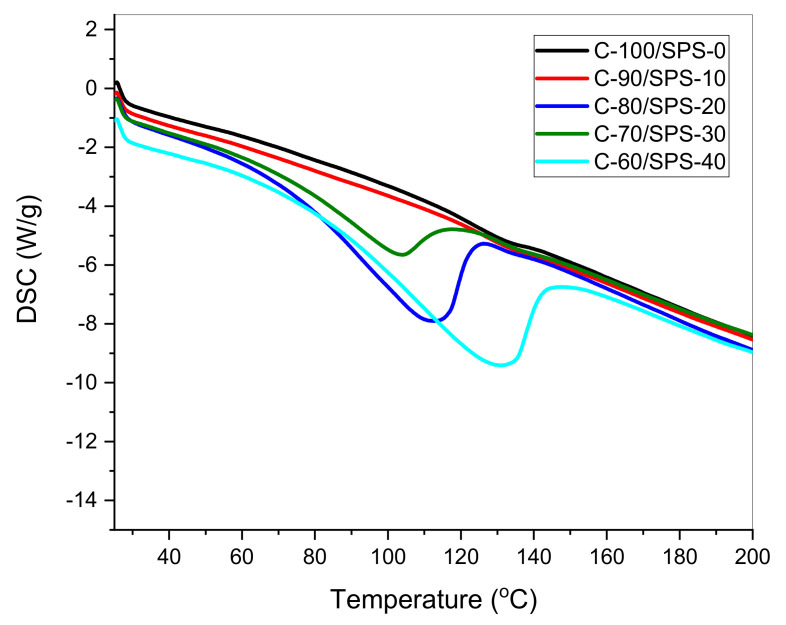
DSC curves for char briquettes with 0, 10, 20, 30, and 40% SPS loading within the temperature range of 25–600 °C.

The DSC curves illustrated in Figure 10 explain the heat flows of char briquettes with 0, 10, 20, 30, and 40% SPS loading, associated with thermal decomposition. In addition, the DSC curves provide details about the changes in enthalpy and the onset temperature of physical and chemical changes. Thus, as shown in Figure 10, the DSC curves indicate that there are endothermic (heat absorption) peaks at 100–140 °C under a deoxygenated environment. The endothermic peak attributed to the melting point of the char briquettes varied in the range of 100–140 °C, whereas no clear peak showed the decomposition temperature of the composites. Thus, there is no conclusive decomposition temperature, T_d_. This is due to the absence of exothermic or endothermic peaks. These results are supported by previous works [15,21,27].

### 3.5. Higher Heating Value (HHV) Analysis

The higher heating values of the briquettes are represented in Table 4. When compared with the C-100/SPS-0 briquette and others, the higher heating values show a substantial decrease—except for C-80/SPS-20, with approximately 2% performance enhancement recorded. Generally, the HHVs of samples decrease along with the increase in SPS content, which is in accordance with the literature [19,21,22]. Previous work on biomass waste briquettes found that they exhibited higher HHVs than our plastic waste briquettes, such that orange bagasse charcoal briquettes obtained approximately 27 kJ/g [19] and sugarcane bagasse charcoal approximately 28 kJ/g of HHV [22]. Thus, C-80/SPS-20, with almost 18 MJ/kg, shows a promising future as a charcoal for domestic fuel applications.

### 3.6. Density and Compressive Strength

Table 5 displays the density values of the char briquette biocomposites, and the results of their compressive strength from their resistance to the compression test. From the density results, there are slight increases in the density values of the briquettes comprised of 0, 10, 20, 30, and 40%. According to Demirbas and Sahin-Demirbas [46], density is a paramount characteristic to be taken into account in order to evaluate the quality of the char. The higher the density value, the higher the energy/volume ratio of the briquettes. Thus, in term of energy/volume ratio, C-60/SPS-40 briquettes exhibited the highest value—0.7 g/cm^3^.

**Figure 11:**
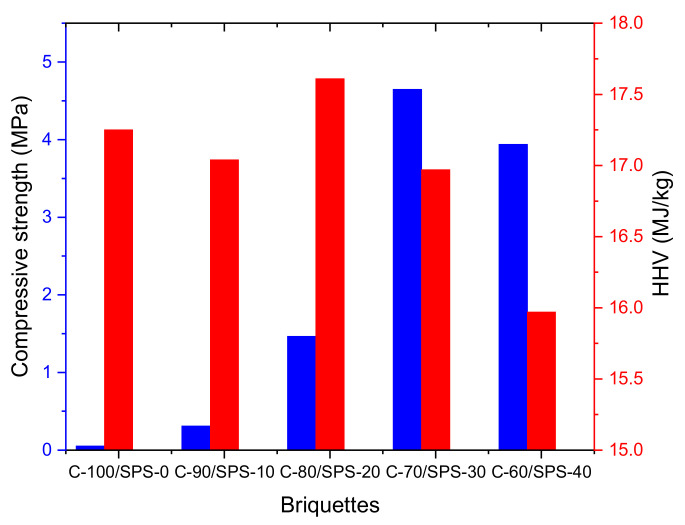
HHV and compressive strength of the char briquettes.

On the other hand, the briquettes with the most resistance to the application of compression were the C-70/SPS-30 briquettes, with 4.65 MPa compressive strength—improved 9194% from the neat char briquettes (0.05 MPa). This rapid enhancement in mechanical properties is due to the loading of starch agglomerates, which bind successfully with the char particles, making them stronger than those made with a lower amount of SPS as a binder. This observation is in keeping with previous works on char briquettes [21,22,29,47]. However, the calorific values (HHV) and mechanical properties of the char briquettes need to be considered in order to select the most suitable briquettes for fuel application and other domestic uses. From Figure 11, we concluded that C-70/SPS-30 briquettes displayed optimal mechanical and combustion properties of 4.647 MPa and 17 MJ/kg, respectively.

## 4. Conclusions

In this study, the elemental, mechanical, and HHV results indicated that PP waste has the potential to be utilized for charcoal briquette production with the aid of sugar palm starch (SPS) as a binder. Slow pyrolysis was carried out on PPE waste, with a final temperature of 450 °C, and yielded solid char, which was filled with various percentages of SPS as a binder prior to compression moulding. Thermal analysis indicates that, with increased SPS loading, the weight percentages of fixed carbon decrease due to increased volatile matter. The results of SPS loading on compressive strength indicate that higher binder loading within the biocomposites contributed to improved mechanical properties, whereas the calorific values were reduced. In our study, the biocomposite char briquettes presented excellent compressive strength and high calorific values compared to other previous works on plastic waste. With a high amount of carbon and a high calorific value, the char briquette C-80/SPS-20 showed the best characteristics for use as a domestic and commercial charcoal in the form of solid fuel briquettes.

## Figures and Tables

**Table 1:** Calorific values of other waste-derived briquettes.

Char Briquette	Calorific Value (MJ/kg)	Reference
Sugarcane bagasse	28.32	[22]
Orange bagasse	26.47	[19]
Human waste	25.1	[27]
Groundnut shells	22.50	[28]
Waste plastic and coal	19.27	[15]
Rice husk	17.04	[29]
Rice straw and rice husk ash	17.01	[29]
Paper and saw dust	16.68	[30]
Waste oil	14.65	[31]
Leather cassava tubers and sludge	7.68	[16]
Used COVID-19 polypropylene isolation gown waste	-	Current work

**Table 2:** EDX analysis element content.

Element	Peak (keV)	Weight (%)	Atomic (%)
C K	0.28	31.36	43.28
O K	0.52	44.53	46.14
Al K	1.49	0.55	0.34
P K	2.02	3.57	1.91
S K	2.33	0.49	0.25
K K	3.31	0.37	0.16
Ca K	3.63	19.13	7.91
Total		100.00	

**Table 3:** Proximate analysis of the briquettes (wt.%).

Briquette	Moisture	Volatile Matter	Ash	Fixed Carbon
C-100/SPS-0	1.33	4.88	6.37	88.75
C-90/SPS-10	2.36	11.60	7.76	80.64
C-80/SPS-20	18.53	15.34	5.85	78.81
C-70/SPS-30	36.52	10.36	5.01	84.63
C-60/SPS-40	44.67	15.23	5.26	79.51

**Table 4:** Higher heating values (HHVs).

Briquettes	HHV (J/g)
C-100/SPS-0	17,251.96
C-90/SPS-10	17,041.92
C-80/SPS-20	17,614.30
C-70/SPS-30	16,969.61
C-60/SPS-40	15,967.31

**Table 5:** Density and compressive strength of the char briquettes.

Briquettes	Density (g/cm^3^)	Yield Strength (MPa)	Compressive Strength (MPa)
C-100/SPS-0	0.541 ^a^ ± 0.010	0.037 ^a^ ± 0.032	0.050 ^a^ ± 0.010
C-90/SPS-10	0.539 ^a^ ± 0.023	0.177 ^a^ ± 0.035	0.310 ^a^ ± 0.066
C-80/SPS-20	0.601 ^b^ ± 0.020	1.337 ^b^ ± 0.515	1.463 ^b^ ± 0.424
C-70/SPS-30	0.673 ^c^ ± 0.019	4.380 ^c^ ± 0.686	4.647 ^c^ ± 0.779
C-60/SPS-40	0.700 ^c^ ± 0.033	3.700 ^c^ ± 0.161	3.937 ^c^ ± 0.038

^a, b, c^ values with different letters in the same column are significantly different (*p* < 0.05).

## Data Availability

The data presented in this study are available on request from the corresponding author.
